# Development and external validation of an AI pipeline for automated estimation of a radiographic DMFT (rDMFT) score from panoramic radiographs: a feasibility study

**DOI:** 10.1007/s00784-026-07214-x

**Published:** 2026-10-15

**Authors:** Babak Saravi, Veronika Shavlokhova, Ashraf Türky, Daman Deep Singh, Felix Schrader, Andreas Vollmer, Norbert Kübler, Christoph Sproll, Lara Schorn

**Affiliations:** 1 Department of Oral, Maxillofacial and Facial Plastic Surgery, Medical Faculty and University Hospital Düsseldorf, Heinrich-Heine-University Düsseldorf, Düsseldorf, 40225 Germany; 2 Department of Oral and Maxillofacial Surgery, University Clinic Ruppin-Brandenburg, Neuruppin, 16816 Germany; 3 Department of Oral and Maxillofacial Plastic Surgery, University Hospital of Würzburg, Würzburg, 97070 Germany

**Keywords:** Artificial intelligence, Deep learning, Panoramic radiography, Dental caries, Radiographic DMFT (rDMFT), External validation

## Abstract

**Objectives:**

To assess the feasibility of estimating a radiographic Decayed-Missing-Filled-Teeth score (rDMFT), a proxy for the clinical DMFT index, from a single panoramic radiograph with a position-aware deep-learning pipeline.

**Materials and methods:**

A tooth-status detector and a tooth-numbering detector (32 FDI positions), trained on DENTEX 2023, were combined in a position-aware read-out (one tooth per FDI position, third molars excluded, missing teeth = 28 − occupied positions) and validated on 100 panoramic radiographs from an independent hospital against a two-dentist, radiograph-based reference (intraclass correlation coefficient, ICC; mean absolute error, MAE; Bland–Altman analysis).

**Results:**

Against the reference, the pipeline reached an ICC of 0.84 (95% CI 0.74 to 0.90) and an MAE of 2.38, below the between-dentist agreement in point estimate (ICC 0.88, MAE 2.13), with larger errors against each dentist alone (MAE 3.00 and 2.73) and a bias of + 1.14 units from caries over-detection; agreement was highest for filled (r 0.90) and lowest for decayed teeth (r 0.34; dentists 0.16). In the 26 radiographs with missing teeth the missing count was exact in 42% (62% within ± 1 tooth), and missing teeth were over-called in 15 of 74 complete dentitions; tooth loss was no longer overlooked (26 of 26 detected), but the overall ICC was 0.84 versus 0.90 without numbering.

**Conclusions:**

Automated rDMFT estimation is feasible, but agreement was lower in point estimate than between two dentists, with larger errors against each dentist. The missing component now responds to actual tooth loss but remains imprecise and is over-called in some complete dentitions; the decayed component is unreliable. The pipeline is a research building block, not a decision-support or triage tool.

**Clinical relevance:**

rDMFT is a radiographic proxy, not a clinical measure, and the pipeline is not proposed for clinical use; any application would first require validation in unselected cohorts against clinically determined reference standards.

**Supplementary Information:**

The online version contains supplementary material available at https://doi.org/10.1007/s00784-026-07214-x.

## Introduction

Untreated caries in the permanent dentition remains the most prevalent chronic condition in the Global Burden of Disease estimates, affecting about 2.3 billion people [1, 2], and oral diseases rank among the most costly and under-served non-communicable diseases [3]. The Decayed-Missing-Filled-Teeth (DMFT) index has been the standard epidemiological summary of lifetime caries experience since the 1930s and remains the dominant measure in adult-population studies [4, 5].

The DMFT index is an epidemiological instrument established by clinical examination, supplemented where available by radiographs; it is not routinely calculated as a patient-level score in everyday care. Panoramic radiographs (orthopantomograms, OPGs) provide the only single-acquisition view of the full dentition but cannot establish why a tooth is missing or was restored and resolve many carious lesions poorly, so counts read from an OPG are a radiographic proxy for caries experience, themselves subject to inter-rater variability [6]. Large archives of standardised OPGs and mature deep-learning methods [7] nevertheless make the automated estimation of such a proxy a natural feasibility question.

Reviews of artificial intelligence in dental radiology document rapid progress on adjacent tasks — caries detection [8, 9], periodontal bone-loss assessment [10, 11], restoration detection and charting [12, 13], and tooth detection and FDI numbering [14] — and characterise the architectures in use [15, 16]. The public DENTEX 2023 dataset provides a shared benchmark of 1,005 hierarchically annotated OPGs [17], and recent work extends restoration detection to caries under fixed prostheses [18].

External validation of dental-AI systems on institutionally independent cohorts remains uncommon [13], and performance is seldom benchmarked against per-patient endpoints with the uncertainty quantification required by current reporting frameworks [19, 20]. To the best of our knowledge, no deep-learning system for automated rDMFT estimation from panoramic radiographs has yet been published. Here we report a feasibility study of a pipeline that combines tooth-status detection with tooth numbering, so that decayed, missing and filled teeth are counted per FDI position, validated externally against a two-dentist reference. The estimated quantity is termed radiographic DMFT (rDMFT) throughout; DMFT denotes only the clinical-epidemiological index.

## Materials and methods

### Datasets

#### Training and internal validation data 

Both detectors were trained on the public DENTEX 2023 dataset [17], which provides anonymised OPGs from three European hospitals in two partly overlapping partitions: 705 radiographs with human bounding-box annotations of abnormal teeth (caries, deep caries, periapical lesion, impacted tooth) and a quadrant–enumeration partition of 634 radiographs in which every tooth carries its FDI position (260 radiographs occur in both, under different file names). For the tooth-status detector, the 705 radiographs were re-coded into three mutually exclusive classes (20,699 boxes; Table S8): Caries (both DENTEX caries grades), Filled (restorations from a list compiled by one study dentist and mapped by FDI position, taking precedence over a caries annotation on the same tooth) and Healthy (all remaining present teeth, irrespective of impaction or periapical status, with boxes taken from the dataset’s per-tooth enumeration layer). The restoration list recorded restored versus not restored only and was not independently verified. The 705 radiographs were split image-wise 80/20 (564 training, 141 validation; seed 42); the numbering detector used the same split ratio on the enumeration partition (506 training; 123 validation after excluding five radiographs pixel-identical to a training image). Because the partitions overlap (35 of the 123 had been used to train the status detector), the numbering validation split served only for the numbering detector and its confidence threshold. 

#### External validation cohort

From a series of 269 consecutively acquired OPGs extracted in an earlier project at the Department of Oral, Maxillofacial and Facial Plastic Surgery of University Hospital Düsseldorf, 100 anonymised radiographs were drawn at random for external validation. The selected radiographs were re-identified with blinded codes D001 – D100 in randomised order prior to annotation.

### Reference standard for the external cohort

Two dentists (raters A and B), oral-and-maxillofacial surgeons with the German dual qualification in dentistry and medicine and at least five years of panoramic-radiograph experience, annotated the 100 blinded OPGs independently after aligning their scoring on a shared calibration set, following a written manual: per OPG, the integer counts of decayed (D), missing (M) and filled (F) permanent teeth on a 28-tooth base (third molars excluded) and a self-reported confidence rating (1 = low to 3 = high). They received the OPGs in the same randomised order and were blind to each other’s annotations and to all AI outputs; no OPG was excluded. The reference rDMFT was the component-wise mean of the two raters, rounded to the nearest integer (ties to even), with rDMFT = D + M + F; being derived from the radiographs, it is a reference rDMFT, not a clinical DMFT.

### Detection pipeline and position-aware read-out

Tooth status was detected with a YOLO26x detector (Ultralytics, Frederick, Maryland, USA), an end-to-end single-stage detector without non-maximum suppression, pretrained on COCO and fine-tuned on the three status classes [21] (AdamW, initial learning rate 1 × 10^− 3^, cosine schedule, image size 1024 pixels, mosaic augmentation, early stopping with a patience of 20 epochs on validation mean Average Precision; 67 epochs). A second YOLO26x detector was trained for tooth numbering on the 32 FDI positions with the same recipe, except that horizontal-flip augmentation was disabled because a flipped radiograph swaps the left and right quadrants (50 epochs). Training and inference ran on one NVIDIA GeForce RTX 3090 GPU (NVIDIA Corporation, Santa Clara, California, USA).

At inference, the status detector was applied with four geometric augmentations (identity, horizontal flip, scales 0.83× and 1.20×) [22, 23], and the back-projected predictions of the four passes were fused by weighted boxes fusion at an intersection-over-union (IoU) threshold of 0.55 [24] with per-class confidence thresholds of 0.30 (Healthy, Caries) and 0.45 (Filled), fixed on the internal validation split. The numbering detector was applied in a single pass. The position-aware read-out then applied rules fixed in advance: (i) numbering boxes with confidence ≥ τ were processed in descending confidence, and a box was retained if its FDI position was still unassigned and it did not overlap (IoU > 0.5) an already retained box, so that each position held at most one tooth and no tooth occupied two positions; (ii) the third-molar positions (18, 28, 38, 48) and the status boxes overlapping them were excluded, giving the 28-tooth base of the reference; (iii) each occupied position took the status of the overlapping status boxes (IoU ≥ 0.5) with the precedence Filled > Caries > Healthy used for the training labels, an occupied position without a status box counting as present; (iv) status boxes overlapping no retained position were not counted; (v) M = 28 − occupied positions, D and F = positions with Caries and Filled status, and rDMFT = D + M + F. Because every tooth is counted once at one position, the present count cannot exceed 28. The threshold τ was selected on the numbering validation split by minimising the mean absolute error of the present-tooth count against the human labels (23 candidates; Table S7) and applied unchanged to the external cohort. Two further read-outs were evaluated externally for comparison only: the count-based read-out of the pipeline’s initial version (no numbering; M = max(0, 28 − detected teeth)) and a sensitivity variant counting status boxes matched to no position as present teeth of unknown position (capped at 28). The position-aware read-out was designated primary on construct-validity grounds — each component is traceable to tooth positions — after the count-based read-out had already been evaluated on this cohort, so the comparison between read-outs is descriptive.

### Evaluation metrics and statistical analysis

Both detectors were characterised on their validation splits by per-class Average Precision (AP, IoU 0.50:0.95), AP50, AP75, precision and recall (COCO conventions); for the numbering detector, the precision and recall of the occupied non-third-molar positions against the human labels and the per-image error of the present-tooth count were added. The contribution of test-time augmentation (TTA) was assessed by the internal count error and by the external rDMFT agreement with and without TTA.

The primary external endpoint was the agreement between the AI-estimated and the reference rDMFT per OPG: Bland–Altman bias and 95% limits of agreement (LoA) [25], Pearson r, ICC (2,1) (two-way random effects, absolute agreement, single measurement, treating AI and reference as two interchangeable raters), MAE, and the cumulative agreement (fraction of OPGs within N units, *N* = 0 to 8), overall and per component. Because the difference may depend on the magnitude of the score, it was regressed on the mean of the two measurements, and regression-based LoA, with the residual spread modelled as a linear function of the mean, are reported alongside the conventional limits. The AI was also compared with each rater individually, and differences between the AI-to-reference or AI-to-rater and the inter-rater ICC and MAE, and between the decayed-component correlations, were estimated by paired bootstraps. Inter-rater reliability was characterised by ICC (2,1) and linear-weighted Cohen’s κ [26, 27]. For the missing component, the detection of any tooth loss was summarised as sensitivity and specificity with Wilson 95% confidence intervals (CIs), agreement was examined within the subset with missing teeth, and the read-outs were compared by paired bootstraps of the differences in MAE and ICC. Compensation between component errors was quantified as the share of OPGs with all three components within ± 1 unit and the share in which a total error of ≤ 1 unit concealed a component error of ≥ 2 units.

Subgroup analyses, defined before the external analysis, were performed by reference rDMFT severity (0, 1–5, 6–12, ≥ 13), by rater A’s self-reported confidence and by image-sharpness tercile (Laplacian variance). All analyses were performed in Python 3.10 (Python Software Foundation, Wilmington, Delaware, USA) with percentile-bootstrap CIs (10,000 resamples, seed 42, throughout). ICC values between 0.75 and 0.90 were interpreted as good and values above 0.90 as excellent [28].

### Ethics and reporting standards

Use of the DENTEX 2023 dataset was governed by its CC-BY research licence. Use of the 100 Düsseldorf OPGs was approved by the Ethics Committee of the Medical Faculty of University Hospital Düsseldorf (reference number 2024–3049). All radiographs were anonymised prior to transfer and analysis. Reporting follows the CLAIM checklist for medical AI publications (Table S3) and the relevant items of the TRIPOD + AI statement.

## Results

### Study cohort

**Fig. 1:**
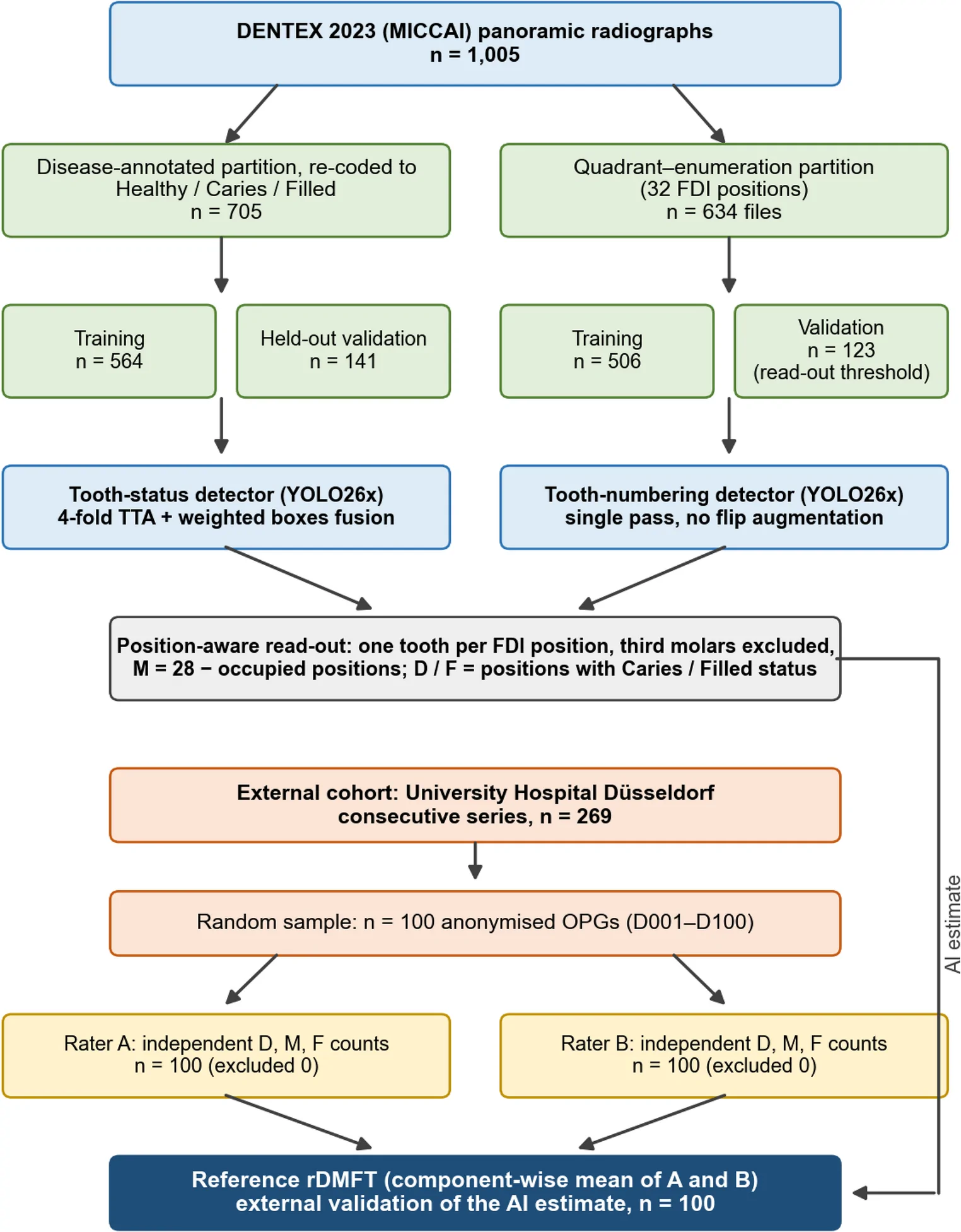
Study flow diagram. The tooth-status detector was trained on the 705 disease-annotated radiographs of the public DENTEX 2023 dataset (564 training / 141 held-out validation) and the tooth-numbering detector on the dataset’s 634-radiograph enumeration partition (506 training / 123 validation); the read-out threshold was selected on the numbering validation split. External validation used 100 anonymised OPGs drawn at random from 269 consecutively acquired OPGs at University Hospital Düsseldorf, with reference D, M and F counts produced independently by two dentists and adjudicated by component-wise averaging

The flow of OPGs through training, internal validation and external validation is shown in Fig. 1. The external cohort comprised 100 anonymised OPGs with a reference rDMFT spanning the full range (median 4, range 0–28); missing teeth were uncommon (74 OPGs had none; mean M 1.31, maximum 19).

### Internal performance of the two detectors

**Fig. 2:**
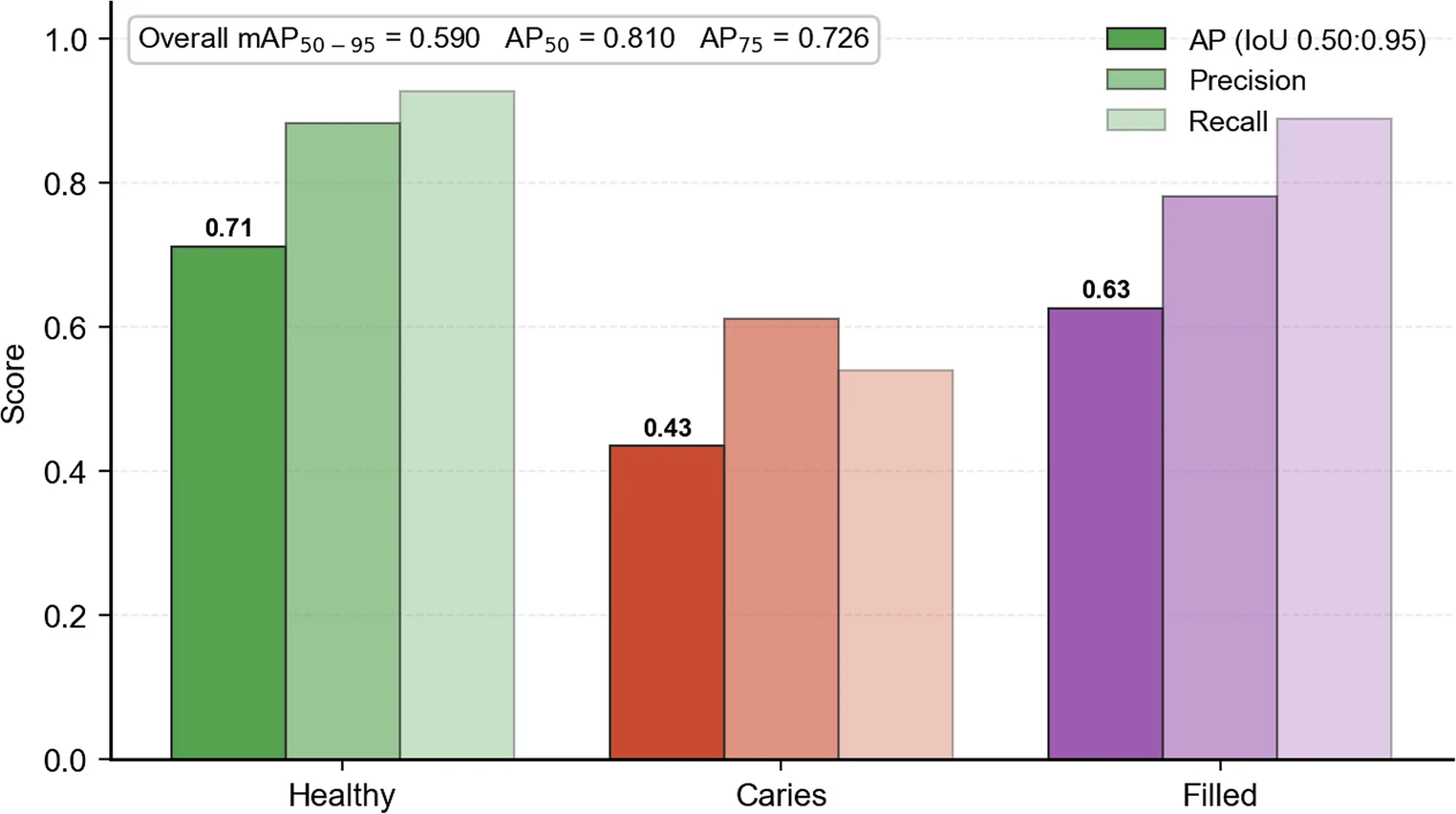
Per-class detection performance on the held-out internal validation set (*n* = 141): Average Precision (IoU 0.50:0.95), precision and recall for the Healthy, Caries and Filled classes. Caries is the most difficult class (AP 0.43)

On its held-out split (*n* = 141), the status detector achieved an overall AP of 0.59 (AP50 0.81, AP75 0.73, precision 0.76, recall 0.79; Fig. 2; Table S1), strongest for Healthy (AP 0.71) and weakest for Caries (AP 0.43). The numbering detector reached a precision of 0.91, a recall of 0.92 and a mAP50 of 0.94 on its 123 validation radiographs (Table S6); at the selected threshold (τ = 0.05), the occupied positions matched the human labels with a precision of 0.985 and a recall of 0.984, and the present-tooth count was exact in 74% and within ± 1 tooth in 91% of radiographs (Table S7). Four-fold TTA reduced the status detector’s internal count error (4.19 vs. 4.40) and was retained; without TTA the external rDMFT MAE was 2.27 instead of 2.38.

### Inter-rater reliability of the external reference standard

The two dentists agreed strongly on the per-OPG reference rDMFT (Table 1): *r* = 0.911, ICC (2,1) = 0.876 and linear-weighted κ = 0.704, in the band described by Landis and Koch as substantial (κ 0.61–0.80) [29] and within the good ICC band [28]. Agreement differed sharply by component (Table S4, Fig. S4): near-perfect for missing (*r* = 0.995, κ 0.95) and filled teeth (*r* = 0.992, κ 0.94), but close to chance for decayed teeth (*r* = 0.16, 95% CI 0.00 to 0.32; κ 0.18), so caries scoring was poorly reproducible even between two experienced dentists.

**Table 1:** Inter-rater reliability of the two dentists on the external Düsseldorf cohort (*n* = 100 OPGs; per-OPG reference rDMFT). The raters were blind to each other and to the AI estimates

Statistic	Value
Pearson r (rater A vs. rater B)	0.911
ICC (2,1)	0.876
Linear-weighted Cohen’s κ	0.704
Mean absolute error (rDMFT units)	2.13

### External validation against the reference rDMFT

**Fig. 3:**
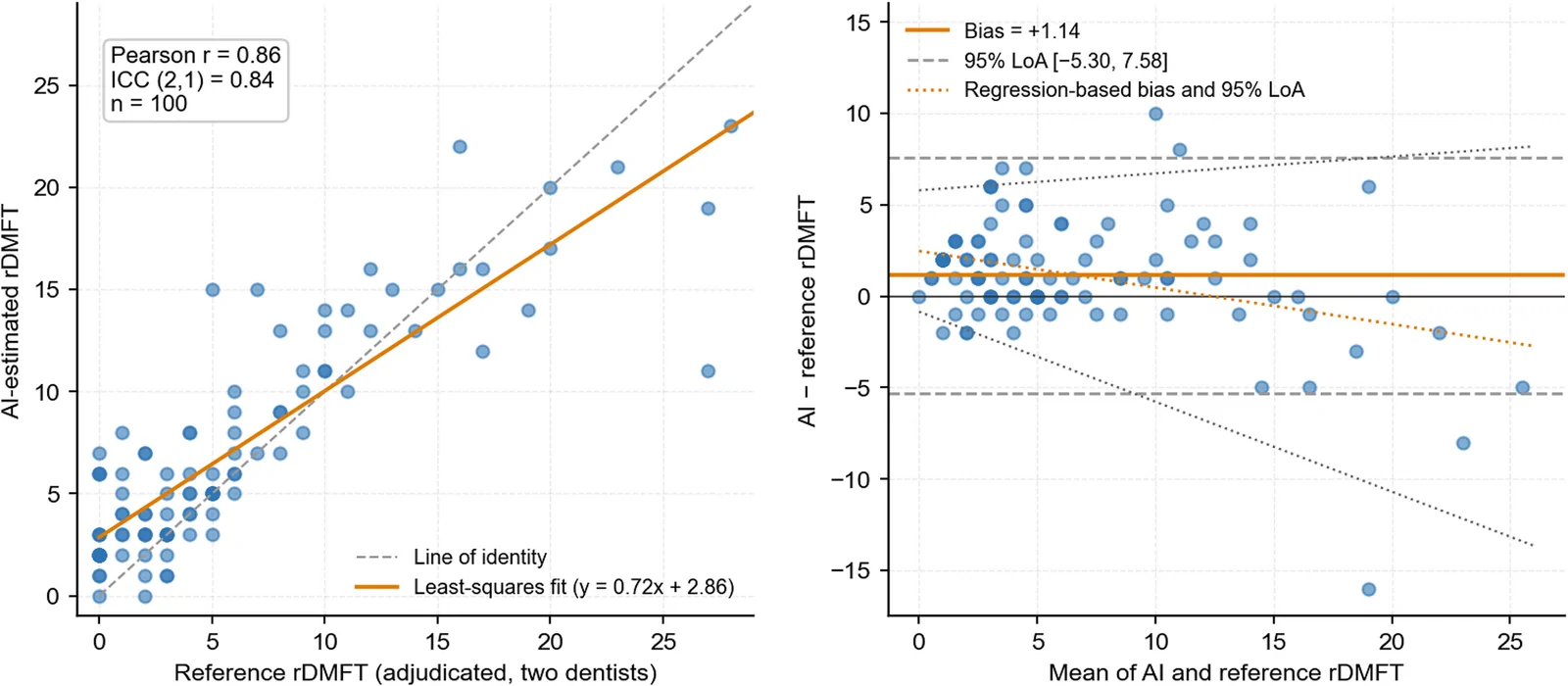
AI-estimated versus reference rDMFT on the external Düsseldorf cohort (*n* = 100). Left: scatter plot with line of identity (grey dashed) and least-squares fit (orange). Right: Bland–Altman plot with mean bias (solid line) and conventional 95% limits of agreement (dashed lines); dotted lines show the regression-based bias and limits, which depend on the magnitude of the score

Against the reference rDMFT, the pipeline reached *r* = 0.86 (95% CI 0.78 to 0.92) and an ICC of 0.84 (95% CI 0.74 to 0.90; Fig. 3; Table 2), versus an ICC of 0.88 between the two dentists (paired difference − 0.04, 95% CI − 0.14 to 0.06). Against each dentist alone, agreement was lower (rater A: ICC 0.81, MAE 3.00; rater B: ICC 0.80, MAE 2.73) than against the averaged reference, as expected because averaging reduces rater noise, and both errors exceeded the between-dentist MAE of 2.13 (paired differences + 0.87, 95% CI 0.20 to 1.58, and + 0.60, 95% CI − 0.06 to 1.28). The estimate was systematically high (bias + 1.14 units, 95% CI 0.47 to 1.76; 95% LoA − 5.30 to 7.58; MAE 2.38, 95% CI 1.91 to 2.90). The difference decreased with the magnitude of the score (slope − 0.20 per unit, 95% CI − 0.35 to − 0.03) while its spread increased, so regression-based LoA are also given: −1.83 to 5.97 at a mean score of 2, − 5.77 to 6.71 at 10 and − 10.69 to 7.64 at 20 (Table 2).

**Table 2:** Agreement between the AI estimate (position-aware read-out) and the reference rDMFT on the external Düsseldorf cohort (*n* = 100 OPGs). 95% CIs are percentile-bootstrap intervals (10,000 resamples); bias, LoA and MAE are in rDMFT units; component biases are AI minus reference. Regression-based LoA model the bias and the residual spread as linear functions of the mean of AI and reference

Metric	Value	95% CI
Pearson r (rDMFT)	0.86	0.78, 0.92
ICC (2,1) (rDMFT)	0.84	0.74, 0.90
MAE (rDMFT units)	2.38	1.91, 2.90
Bland–Altman bias (rDMFT units)	+ 1.14	0.47, 1.76
Bland–Altman 95% LoA	−5.30, 7.58	—
Pearson r — Decayed (D)	0.34	0.09, 0.53
Pearson r — Missing (M)	0.83	0.67, 0.91
Pearson r — Filled (F)	0.90	0.84, 0.94
MAE — Decayed (D)	1.75	1.49, 2.03
MAE — Missing (M)	0.92	0.58, 1.31
MAE — Filled (F)	1.15	0.84, 1.49
Bias — Decayed (D)	+ 0.83	0.42, 1.23
Bias — Missing (M)	+ 0.30	−0.12, 0.70
Bias — Filled (F)	+ 0.01	−0.39, 0.39
Slope of the difference on the mean (per rDMFT unit)	−0.20	−0.35, − 0.03
Regression-based 95% LoA at a mean score of 2 / 10 / 20	−1.83, 5.97 / −5.77, 6.71 / −10.69, 7.64	—

### Cumulative agreement

**Fig. 4:**
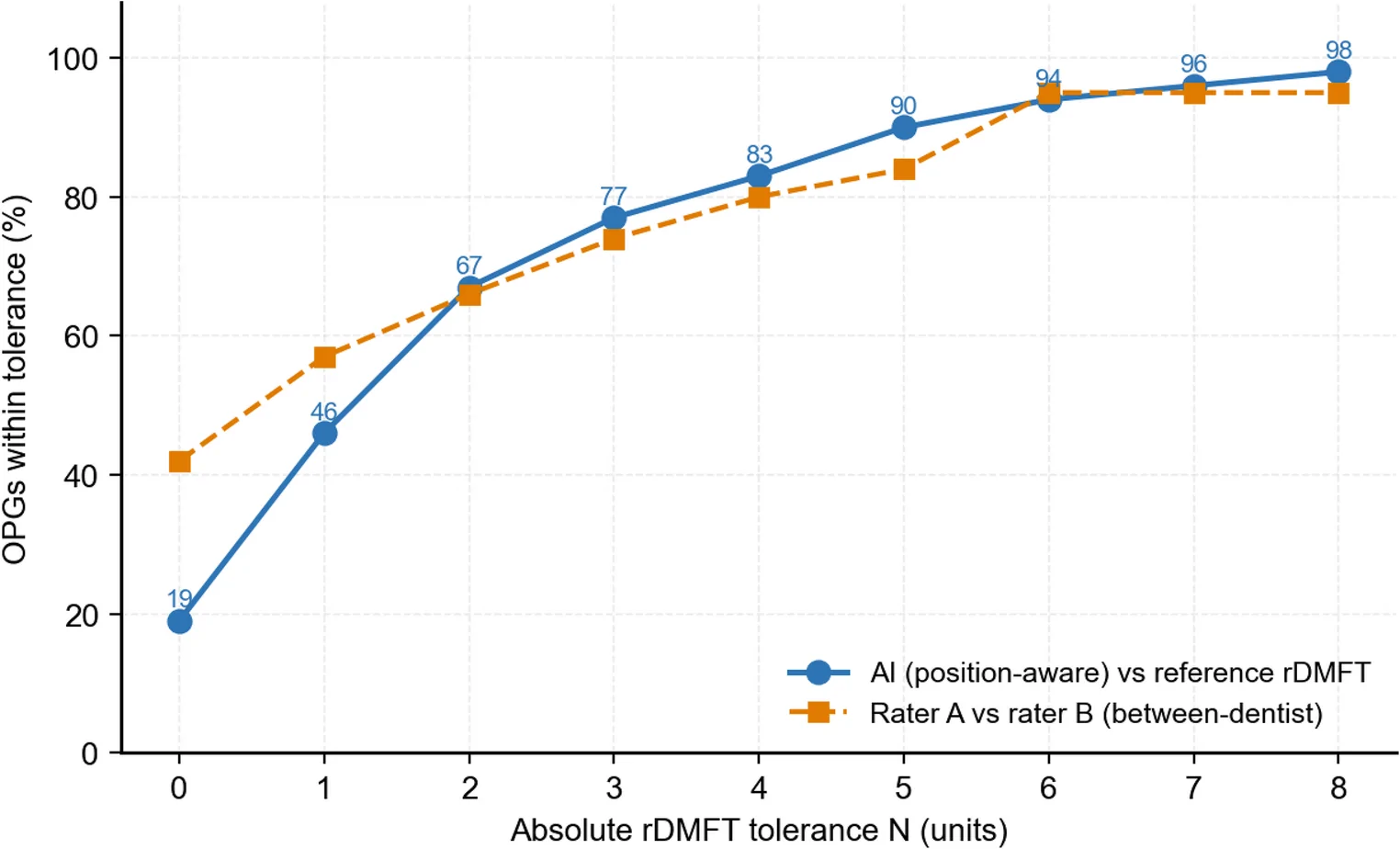
Cumulative agreement on the external Düsseldorf cohort. Blue: fraction of OPGs in which the AI-estimated rDMFT lies within a given absolute tolerance N of the reference. Orange: fraction in which the two dentists lie within N of each other. Values are listed in Table S2

Of the 100 OPGs, 46% were within ± 1 rDMFT unit of the reference, 67% within ± 2, 77% within ± 3 and 90% within ± 5 (Fig. 4; Table S2). The two dentists agreed more often at the strictest tolerances (42% vs. 19% exact; 57% vs. 46% within ± 1) and similarly at ± 3 (74% vs. 77%).

### Component-wise performance

Per component (Fig. S1; Table 2), agreement was highest for filled teeth (*r* = 0.90, MAE 1.15, bias + 0.01), intermediate for missing teeth (*r* = 0.83, MAE 0.92, bias + 0.30) and weak for decayed teeth (*r* = 0.34, MAE 1.75, bias + 0.83), whose over-detection accounts for most of the total bias. The pipeline’s caries agreement (*r* = 0.34, 95% CI 0.09 to 0.53) and the between-dentist agreement (*r* = 0.16, 95% CI 0.00 to 0.32) had overlapping confidence intervals (paired Δr = + 0.17, 95% CI − 0.11 to 0.41); neither supports radiograph-only caries scoring [9].

### Missing component under position-aware counting

The numbering detector assigned a mean of 29.6 positions per OPG, including 3.25 third-molar positions that were excluded, and a mean of 0.24 status boxes per OPG matched no retained position. The present-tooth count never exceeded 28 (mean 26.4; range 6–28) and showed no systematic bias against the reference (− 0.30 teeth, 95% CI − 0.70 to 0.12; 70% exact, 79% within ± 1 tooth), whereas the raw number of status boxes exceeded 28 in 84 of 100 OPGs (maximum 38) and still did so in 77 after class-agnostic removal of overlapping boxes: detected third molars, not duplicate detections, were the main reason the count-based read-out favoured M = 0. Missing teeth were uncommon in the reference (26 of 100 OPGs). Within these 26, the pipeline detected tooth loss in all (sensitivity 100%, 95% CI 87% to 100%), but the missing count was exact in only 42% and within ± 1 tooth in 62% (MAE 1.96, 95% CI 1.00 to 3.08). Conversely, missing teeth were over-called in 15 of the 74 OPGs without missing teeth (specificity 80%, 95% CI 69% to 87%; 41 false missing teeth, one to four per OPG), predominantly at premolar positions (30 of 41): at 37 of these positions the numbering detector had placed the box of a neighbouring position on the same tooth, so that the second label was discarded by the one-tooth-per-position rule, and at 4 it produced no box above the threshold. Over-calls in complete dentitions and under-counts in dentitions with tooth loss (120 of 131 missing teeth estimated) offset each other, so the net bias of the missing component is small (+ 0.30) despite its MAE of 0.92.

### Comparison of read-outs

Table 3 compares the read-outs. Relative to the count-based read-out, the position-aware read-out had a lower ICC point estimate (0.84 vs. 0.90; paired difference − 0.06, 95% CI − 0.15 to 0.00) and a higher MAE (2.38 vs. 2.21; +0.17, 95% CI − 0.19 to 0.60), wider limits of agreement and fewer OPGs within ± 3 units (77% vs. 84%); the decayed and filled errors were slightly lower and the missing error higher (0.92 vs. 0.65). The lower missing error of the count-based read-out reflects its structural preference for M = 0: it assigned M = 0 in 86 OPGs, detected tooth loss in only 13 of the 26 affected OPGs (8% exact, 35% within ± 1 tooth) and over-called it in 1, so its higher overall correlation does not indicate better missing-tooth assessment. The sensitivity variant changed the results little (ICC 0.83; MAE 2.39; tooth loss detected in 24 of 26; over-called in 14 of 74), and the results were stable across the flat region of the internal selection criterion (τ = 0.01 to 0.15: ICC 0.80–0.84, MAE 2.38–2.49, tooth loss detected in 26 of 26 throughout; Table S7). Component errors partly cancelled: all three components were within ± 1 unit in only 29% of OPGs, and in 21% a total error of ≤ 1 unit concealed a component error of ≥ 2 units, so agreement on the total does not certify tooth-level accuracy.

**Table 3:** Comparison of read-outs on the external Düsseldorf cohort (*n* = 100 OPGs). The count-based read-out uses the same status detections without tooth numbering (M = max(0, 28 − detected teeth)); the sensitivity variant additionally counts status boxes matched to no FDI position as present teeth of unknown position. Sensitivity and specificity refer to the detection of any tooth loss per OPG (Wilson 95% CIs); other CIs are percentile bootstraps (10,000 resamples). Paired differences between read-outs are given in the text

Metric	Count-based, no numbering (ablation)	Position-aware (primary)	Position-aware, unmatched boxes counted (sensitivity)
Pearson r (95% CI)	0.91 (0.87, 0.95)	0.86 (0.78, 0.92)	0.86 (0.78, 0.92)
ICC (2,1) (95% CI)	0.90 (0.83, 0.93)	0.84 (0.74, 0.90)	0.83 (0.74, 0.90)
MAE (95% CI)	2.21 (1.85, 2.60)	2.38 (1.91, 2.90)	2.39 (1.91, 2.91)
Bland–Altman bias	+ 1.25	+ 1.14	+ 1.11
95% LoA	−3.95, 6.45	−5.30, 7.58	−5.40, 7.62
OPGs within ± 1 / ±3 units	42% / 84%	46% / 77%	45% / 77%
MAE decayed / missing / filled	1.90 / 0.65 / 1.20	1.75 / 0.92 / 1.15	1.71 / 0.92 / 1.16
OPGs assigned M = 0 (reference: 74)	86	59	62
Tooth loss detected, of 26 OPGs with missing teeth (sensitivity; 95% CI)	13 (50%; 32–68)	26 (100%; 87–100)	24 (92%; 76–98)
Missing teeth over-called, of 74 OPGs without (specificity; 95% CI)	1 (99%; 93–100)	15 (80%; 69–87)	14 (81%; 71–88)
M exact / within ± 1 in the 26 OPGs with missing teeth	8% / 35%	42% / 62%	42% / 65%
MAE of M in the 26 OPGs with missing teeth (95% CI)	2.46 (1.69, 3.42)	1.96 (1.00, 3.08)	2.04 (0.92, 3.38)

### Subgroup robustness

Subgroup analyses defined before the external analysis (Fig. S2; Table S5) gave MAEs of 2.94 (95% CI 2.06 to 3.94) at rDMFT = 0 (*n* = 17), 1.89 (1.33 to 2.53) at 1–5 (*n* = 45), 2.04 (1.33 to 2.83) at 6–12 (*n* = 24) and 3.86 (1.93 to 6.22) at ≥ 13 (*n* = 14), and 2.15, 2.78 and 2.16 across the lower, middle and upper image-sharpness terciles; the error was highest at both ends of the range, with overlapping confidence intervals, and showed no monotonic dependence on sharpness. The low-confidence stratum (*n* = 3) is listed for completeness only.

### Qualitative examples

Figure S3 shows representative examples with the FDI position assigned to each counted tooth: a caries-free mouth (D003), a mid-range case (D004), a dentition with several missing teeth (D086), a complete dentition in which missing teeth were over-called (D001), and the largest under- and over-estimations (D025, D031).

### Inference time

Mean per-image inference time on a single NVIDIA RTX 3090 GPU was 81 ms for the status detector in single-pass mode (95th percentile 132 ms) and 33 ms for the numbering detector; with four-fold TTA of the status detector, the pipeline requires roughly 0.4 s per OPG.

## Discussion

In this feasibility study, a two-detector pipeline with a position-aware read-out estimated rDMFT from a single panoramic radiograph with an ICC of 0.84 and an MAE of 2.38 units against a two-dentist reference, lower in point estimate than the agreement between the dentists (ICC 0.88, MAE 2.13) and with larger errors against each dentist alone; the estimate was systematically high, almost entirely because of caries over-detection. The principal methodological result concerns the missing component. Counting teeth per FDI position removed the structural bias of the count-based read-out, in which detected third molars drove the raw count above 28 in most radiographs, so that M = 0 was assigned by default and tooth loss was overlooked in half of the affected cases. Position-aware counting detected tooth loss in all 26 affected radiographs but estimated the number of missing teeth exactly in fewer than half of them and produced false missing-tooth calls in 15 of 74 complete dentitions, with a lower overall ICC point estimate; the higher correlation of the count-based read-out partly reflected a setting in which a zero estimate was structurally favoured.

Detection-level gains did not translate into endpoint gains: TTA reduced the internal count error but not the external rDMFT error, echoing the gap between detection metrics and clinical utility noted in reviews of dental caries AI [8, 9]; augmentation strategies should be validated on the target endpoint [22, 23], and weighted boxes fusion [24] remains a cheap alternative to multi-architecture ensembles that did not improve the endpoint here.

The reliability of the reference (ICC 0.876; κ 0.704) is consistent with agreement reported for dental staging on panoramic radiographs [6] and bounds what any estimator can demonstrate against it: the two-rater mean has a reliability of about 0.93 (Spearman–Brown), limiting the attainable correlation to roughly 0.97, and to about 0.44 for the decayed component, whose reference is close to noise. The per-OPG component-count convention yields a usable reference for the total and its missing and filled components at low annotation effort, but not for the decayed component, for which modality, rater calibration and protocol are confounded, and it precludes tooth-level evaluation.

Hamdan et al. reported strong restoration detection on panoramic radiographs with a no-code platform [12], Ayhan et al. extended detection to caries under fixed prostheses [18], and Mureșanu et al. reviewed the pitfalls of automated dental-condition detection on panoramic radiographs [13]; none reports a multi-rater external Bland–Altman validation against a per-radiograph rDMFT-type endpoint or integrates tooth numbering into the count. The present pipeline is a feasibility demonstration and does not meet the validation requirements for clinical use set out in dental-AI reviews and reporting guidance [16, 20]. 

### Interpretation of the estimated score 

A panoramic radiograph cannot reveal whether a tooth was lost to caries rather than to periodontal disease, trauma, orthodontic extraction or agenesis, nor whether a restoration was placed for caries, and it under-detects early and approximal lesions. rDMFT therefore reflects radiographically visible tooth status, not caries experience in the clinical-epidemiological sense, and should not be compared with clinically determined DMFT; the same applies to the reference, which was itself derived from the radiographs. The contribution of this study is an externally validated, per-radiograph estimate of that status with quantified uncertainty and a read-out traceable to tooth positions.

Several limitations apply. First, the score is a radiographic proxy and the reference was radiograph-based, so agreement with clinically determined DMFT is unknown; restored teeth with secondary caries count as Filled, and supernumerary teeth are not modelled. Second, the external cohort comes from a single tertiary maxillofacial-surgery department, so generalisability to unselected populations and general practice is unestablished. Third, the reference consists of per-OPG counts: per-tooth accuracy could not be assessed externally, agreement on the total does not imply tooth-level accuracy, and a per-tooth-annotated external cohort with a clinically grounded reference is the necessary next step. Fourth, the caries over-detection reflects both the difficulty of caries assessment on OPGs and the more liberal caries labelling of the training data; the caries read-out is not reliable enough for any use. Fifth, the missing component depends on the numbering detector, whose missed or merged positions are counted as missing, and substantially reduced dentitions were rare (11 OPGs with three or more missing teeth), so performance in reduced or edentulous dentitions is not established. Sixth, the pipeline emits no calibrated probabilities, and the annotation convention did not permit a demographic-fairness audit [19, 20].

Combining OPGs with bitewing imaging would likely narrow the caries gap, since bitewings resolve approximal lesions that the OPG does not; a prospective reader study would quantify effects on reading time and inter-reader agreement; and retrospective rDMFT estimation on archived OPG collections would require validation in representative, non-referral populations before any surveillance use [1].

## Conclusions

An rDMFT score can be estimated automatically from a single panoramic radiograph, but its agreement with a two-dentist reference (ICC 0.84) was lower in point estimate than the agreement between the dentists (0.88), errors against each dentist were larger, and the limits of agreement were wide and score-dependent. Position-aware counting was required for a missing component that responds to actual tooth loss, yet the missing count remains imprecise and is over-called in some complete dentitions; the decayed component is unreliable for dentists and pipeline alike; and rDMFT over-estimates the reference. The score is a proxy for, not a measurement of, clinical caries experience, and the pipeline is a research building block, not a decision-support or triage tool; any clinical or population-level use would require prospective validation in unselected populations against clinically grounded reference standards. 

## Supplementary Information

Supplementary Material 1.

## Data Availability

The DENTEX 2023 dataset is publicly available from the DENTEX challenge organisers (https://dentex.grand-challenge.org/). The complete training, inference, evaluation and figure-generation source code is openly available at https://github.com/Freiburg-AI-Research/automated-dmft-from-opg under the MIT License. The 100 anonymised external panoramic radiographs and the two-rater DMFT annotation files contain potentially identifying clinical imaging data and are therefore not publicly deposited; they can be made available from the corresponding author upon reasonable request and after completion of a data-transfer agreement, subject to the approval of the Ethics Committee of the Medical Faculty of University Hospital Düsseldorf.
