# Supplementary material for: Development and external validation of an AI pipeline for automated estimation of a radiographic DMFT (rDMFT) score from panoramic radiographs: a feasibility study
Source: Clin Oral Investig. 2026 Oct 15;30(11):527. doi: 10.1007/s00784-026-07214-x (PMC13669134; doi:10.1007/s00784-026-07214-x)
Supplement: Supplementary file 1 [file 784_2026_7214_MOESM1_ESM.docx]

**Online Resources (Supplementary Information)**

**Fig. S1. Per-component agreement (Decayed, Missing and Filled) between the AI estimate (position-aware read-out) and the adjudicated reference on the external cohort (n = 100). Each panel shows AI-estimated against reference counts with the line of identity (dashed) and a least-squares fit (solid); r is the Pearson correlation.**

**Fig. S2. Forest plot of the mean absolute error of the AI-estimated rDMFT against the reference on the external cohort, stratified by subgroups defined before the external analysis (rDMFT severity, rater A self-reported confidence, image-sharpness tercile). Markers indicate the mean; horizontal bars indicate 95% percentile-bootstrap confidence intervals (10,000 resamples); the dashed vertical line marks the overall point estimate.**

**Fig. S3. Representative examples from the external cohort (n = 100). Per panel, the status-detector boxes above the deployed thresholds are overlaid on the anonymised OPG (green Healthy, red Caries, purple Filled) together with the FDI position assigned by the tooth-numbering detector (yellow; grey for the third-molar positions, which are excluded from the count); panel titles give the reference rDMFT and the AI estimate. Cases span a caries-free mouth, a mid-range case, a dentition with several missing teeth, a complete dentition in which missing teeth were over-called, and the largest under- and over-estimations (D003, D004, D086, D001, D025, D031).**

**Table S1. Held-out internal validation metrics of the tooth-status detector (n = 141 radiographs, disjoint from training): per-class detection performance without test-time augmentation. Per-class AP is reported alongside overall AP, AP50, AP75, precision and recall (COCO convention).**

| **Metric** | **Healthy** | **Caries** | **Filled** |
| --- | --- | --- | --- |
| AP (IoU 0.50:0.95) | 0.711 | 0.434 | 0.625 |
| Precision | 0.882 | 0.611 | 0.781 |
| Recall | 0.926 | 0.540 | 0.889 |
| Overall (all classes): mAP50–95 = 0.590; AP50 = 0.810; AP75 = 0.726; precision = 0.758; recall = 0.785 |  |  |  |

**Table S2. Cumulative agreement of the AI estimate (position-aware read-out) with the reference rDMFT on the external cohort (n = 100). The third column gives the corresponding between-rater agreement (rater A vs rater B).**

| **Tolerance \|AI − reference\| ≤ N** | **AI vs adjudicated (%)** | **Rater A vs Rater B (%)** |
| --- | --- | --- |
| 0 (exact) | 19% | 42% |
| 1 | 46% | 57% |
| 2 | 67% | 66% |
| 3 | 77% | 74% |
| 4 | 83% | 80% |
| 5 | 90% | 84% |
| 6 | 94% | 95% |
| 7 | 96% | 95% |
| 8 | 98% | 95% |

**Table S3. Checklist for Artificial Intelligence in Medical Imaging (CLAIM), completed for the present study. CLAIM items follow Mongan J, Moy L, Kahn CE. Checklist for Artificial Intelligence in Medical Imaging (CLAIM): A Guide for Authors and Reviewers. Radiol Artif Intell 2020;2(2):e200029. https://doi.org/10.1148/ryai.2020200029.**

| **Section** | **CLAIM item** | **Reported** | **Location in manuscript** |
| --- | --- | --- | --- |
| Title / Abstract | **1.** Identification as a study of AI methodology, specifying the category of technology used (deep learning). | Yes | Title; Abstract |
|  | **2.** Structured summary of study design, methods, results, and conclusions. | Yes | Abstract |
| Introduction | **3.** Scientific and clinical background, including the intended use and clinical role of the AI approach. | Yes | Introduction |
|  | **4.** Study objectives and hypotheses. | Yes | Introduction (final paragraph) |
| Methods — Study design | **5.** Prospective or retrospective study. | Yes | Methods — Datasets; Declarations — Consent to participate (retrospective) |
|  | **6.** Study goal (e.g., model creation, feasibility, external validation). | Yes | Introduction; Methods (feasibility study with external validation) |
| Methods — Data | **7.** Data sources. | Yes | Methods — Datasets (DENTEX 2023; University Hospital Düsseldorf) |
|  | **8.** Eligibility criteria for inclusion of cases. | Yes | Methods — External validation cohort (consecutive acquisition series; 100 drawn at random; no further eligibility restrictions applied) |
|  | **9.** Data pre-processing steps. | Yes | Methods — Detection pipeline |
|  | **10.** Selection of data subsets, if applicable. | Yes | Methods — 100 OPGs randomly selected from a consecutive series of 269 |
|  | 11. Definitions of data elements (rDMFT components D, M, F; FDI numbering). | Yes | Methods — Reference standard (rDMFT components D, M, F; FDI numbering) |
|  | **12.** De-identification methods. | Yes | Methods — Ethics (anonymised prior to analysis) |
|  | **13.** How missing data were handled. | Yes | Methods — no missing data arose: both raters scored all 100 OPGs (the annotation sheet’s exclusion flag was never used); the missing component is 28 minus the occupied FDI positions |
| Methods — Ground truth | **14.** Definition of the reference standard, in sufficient detail to allow replication. | Yes | Methods — Reference standard (two-rater adjudicated reference rDMFT) |
|  | **15.** Rationale for choosing the reference standard. | Yes | Methods — Reference standard; Discussion |
|  | **16.** Source of ground-truth annotations; qualifications and preparation of annotators. | Yes | Methods — two dentists, blinded |
|  | **17.** Annotation tools. | Yes | Methods — structured spreadsheet, WHO-style component convention |
|  | **18.** Measurement of inter-/intra-rater variability; methods to resolve discrepancies. | Yes | Methods/Results — ICC (2,1), linear-weighted κ; component-wise averaging |
| Methods — Data partitions | **19.** Intended sample size and how it was determined. | Partly | Methods — DENTEX split; external n = 100 (randomly selected from a consecutive series of 269; no formal power calculation) |
|  | **20.** How data were assigned to partitions; proportions. | Yes | Methods — 80/20 image-wise split per detector (status 564 / 141; numbering 506 / 123 after exclusion of five duplicates of training radiographs); external cohort held out entirely |
|  | **21.** Level at which partitions are disjoint. | Yes | Methods — image-wise for the internal splits (the two DENTEX partitions overlap in content; the numbering validation split served only for the numbering detector and its threshold; patient-level disjointness not verifiable in the anonymised DENTEX data); institution-level for the external cohort |
| Methods — Model | **22.** Detailed description of the model (inputs, outputs, architecture). | Yes | Methods — Detection pipeline and position-aware read-out; full code released (Data and code availability) |
|  | **23.** Software libraries, frameworks and packages. | Yes | Methods (Ultralytics YOLO26x framework, Python 3.10) |
|  | **24.** Initialization of model parameters (transfer learning). | Yes | Methods — COCO-pretrained, fine-tuned |
| Methods — Training | **25.** Training approach: augmentation, hyperparameters, number of models. | Yes | Methods — Detection pipeline (two detectors: tooth status and tooth numbering; augmentation and hyperparameters given) |
|  | **26.** Method of selecting the final model. | Yes | Methods — early stopping on validation mAP (both detectors); read-out threshold selected on the numbering validation split (Table S7, this document) |
|  | **27.** Details of ensembling techniques, if applicable. | Yes | Methods — four-fold TTA + Weighted-Box-Fusion |
| Methods — Evaluation | **28.** Metrics of model performance. | Yes | Methods/Results — AP, AP50, AP75, precision, recall, position-occupancy precision and recall, ICC, Pearson r, MAE, Bland–Altman |
|  | **29.** Statistical measures of significance and uncertainty (e.g., CIs). | Yes | Methods — 10,000-resample bootstrap 95% CIs |
|  | **30.** Robustness or sensitivity analysis. | Yes | Results — subgroup analyses defined before the external analysis (Fig. S2 and Table S5, this document); read-out comparison and sensitivity variant (Table 3); threshold robustness (Table S7, this document); TTA ablation |
|  | **31.** Methods for explainability/interpretability and how validated. | No | Not performed; the pipeline outputs bounding boxes and count-level scores without saliency-based explanation |
|  | **32.** Validation or testing on external data. | Yes | External Düsseldorf cohort (n = 100) |
| Results | **33.** Flow of cases, using a diagram for inclusion/exclusion. | Yes | Figure 1 (study flow diagram) |
|  | **34.** Demographic and clinical characteristics of cases. | Partly | Image-level characteristics given; patient demographics not available under the per-OPG component-count annotation (Discussion) |
|  | **35.** Performance metrics for the optimal model on all partitions. | Yes | Results; Tables 2–3; Tables S1 and S6 (this document) |
|  | **36.** Estimates of diagnostic accuracy and their precision (95% CIs). | Yes | Results — bootstrap 95% CIs; Bland–Altman limits of agreement |
|  | **37.** Failure analysis of incorrectly classified cases. | Yes | Results/Discussion; Fig. S3 (over- and under-estimation examples, this document) |
| Discussion | **38.** Study limitations, including bias, uncertainty and generalizability. | Yes | Discussion (limitations paragraph) |
|  | **39.** Implications for practice, including intended use/clinical role. | Yes | Discussion; Abstract — Clinical Relevance |
| Other information | **40.** Registration number and name of registry. | N/A | Not a prospective clinical trial; no registration required |
|  | **41.** Where the full study protocol/code can be accessed. | Yes | Data and code availability (GitHub repository) |
|  | **42.** Sources of funding and support; role of funders. | Yes | Declarations — Funding |

**Fig. S4. Component-wise agreement on the external cohort (n = 100). Orange bars: inter-rater Pearson correlation between the two dentists (raters A vs B); blue bars: AI (position-aware read-out) vs the reference. For the decayed component the two dentists agreed at r = 0.16 (95% CI 0.00 to 0.32) and the AI with the reference at r = 0.34 (95% CI 0.09 to 0.53); the confidence intervals overlap and a paired bootstrap of the correlation difference includes zero (Δr = +0.17, 95% CI −0.11 to 0.41). For missing and filled teeth the reference is highly reliable (inter-rater r = 0.99).**

**Table S4. Component-wise agreement on the external Düsseldorf cohort (n = 100 OPGs). Inter-rater columns give the agreement between the two dentists (A vs B); AI columns give the agreement of the position-aware read-out with the adjudicated reference. Values are Pearson r, intraclass correlation ICC(2,1), linear-weighted Cohen’s κ and mean absolute error (MAE), with 95% percentile-bootstrap confidence intervals (10,000 resamples).**

| **Component** | **Inter-rater r** | **Inter-rater ICC** | **Inter-rater κ** | **Inter-rater MAE** | **AI r** | **AI MAE** |
| --- | --- | --- | --- | --- | --- | --- |
| Decayed (D) | 0.16 (0.00–0.32) | 0.11 (0.00–0.26) | 0.18 | 1.93 (1.44–2.44) | 0.34 (0.09–0.53) | 1.75 (1.49–2.03) |
| Missing (M) | 0.995 (0.992–0.999) | 0.995 (0.991–0.997) | 0.95 | 0.11 (0.05–0.18) | 0.828 (0.670–0.911) | 0.92 (0.58–1.31) |
| Filled (F) | 0.992 (0.987–0.995) | 0.990 (0.984–0.994) | 0.94 | 0.25 (0.15–0.36) | 0.897 (0.838–0.943) | 1.15 (0.84–1.49) |

**Table S5. Accuracy of the AI-estimated rDMFT across subgroups defined before the external analysis, on the external cohort (n = 100 OPGs): mean absolute error (MAE) with subgroup size (n) and 95% percentile-bootstrap confidence intervals (10,000 resamples). Confidence intervals overlap substantially across strata; the low-confidence stratum (n = 3) is reported for completeness only and does not support inference. Confidence strata refer to rater A’s self-reported confidence.**

| **Subgroup** | **n** | **MAE (95% CI)** |
| --- | --- | --- |
| rDMFT = 0 | 17 | 2.94 (2.06–3.94) |
| rDMFT 1–5 | 45 | 1.89 (1.33–2.53) |
| rDMFT 6–12 | 24 | 2.04 (1.33–2.83) |
| rDMFT ≥ 13 | 14 | 3.86 (1.93–6.22) |
| Sharpness: lower tercile | 33 | 2.15 (1.52–2.85) |
| Sharpness: middle tercile | 36 | 2.78 (1.92–3.83) |
| Sharpness: upper tercile | 31 | 2.16 (1.39–3.06) |
| Rater confidence: high | 71 | 2.21 (1.76–2.70) |
| Rater confidence: medium | 26 | 2.88 (1.65–4.42) |
| Rater confidence: low | 3 | 2.00 (1.00–3.00) |

**Table S6. Tooth-numbering detector and position occupancy on its validation split (n = 123 radiographs of the DENTEX enumeration partition, no pixel-identical twin in the numbering training split; single-pass inference without augmentation). Detection metrics follow the COCO conventions (32 FDI classes). Occupancy metrics compare the set of occupied non-third-molar positions produced by the read-out at the selected threshold (τ = 0.05) with the human FDI labels; “localised” additionally requires IoU ≥ 0.5 between the retained box and the human box of that position.**

| **Quantity** | **Value** |
| --- | --- |
| Precision (all 32 positions) | 0.909 |
| Recall (all 32 positions) | 0.922 |
| mAP50 | 0.937 |
| mAP50–95 | 0.539 |
| Mean AP50, third-molar positions (18, 28, 38, 48) | 0.892 |
| Mean AP50, other positions | 0.943 |
| Training epochs (best epoch) | 50 (30) |
| Occupancy precision / recall at τ = 0.05 | 0.985 / 0.984 |
| Localised occupancy precision / recall (IoU ≥ 0.5) | 0.941 / 0.940 |
| Present-tooth count: mean absolute error (teeth per radiograph) | 0.44 |
| Present-tooth count: exact / within ±1 tooth | 74.0% / 91.1% |
| Present-tooth count: radiographs over-counted / under-counted (maximum absolute error) | 14 / 18 (5) |
| Single-pass inference time (RTX 3090, 1024 px) | 33 ms (95th percentile 35 ms) |

**Table S7. Selection of the numbering confidence threshold τ on the validation split of the enumeration partition (n = 123). For each candidate value, the read-out (one tooth per FDI position, third molars excluded) was applied to the numbering detections and the occupied positions were compared with the human FDI labels. The selection criterion was the mean absolute error of the present-tooth count (28-tooth base); the selected value (τ = 0.05) is marked, and the criterion is nearly flat between 0.01 and 0.15. The external columns give the agreement of the position-aware read-out with the reference rDMFT on the 100 external OPGs at each τ (ICC (2,1), MAE, radiographs with detected tooth loss among the 26 with missing teeth, and complete dentitions with over-called missing teeth among 74); they were computed after the selection, did not inform it, and are shown as a robustness check.**

| **τ** | **Internal: mean absolute present-count error** | **Internal: exact (%)** | **Internal: within ±1 (%)** | **Internal: occupancy precision / recall** | **External: ICC (2,1)** | **External: MAE** | **External: tooth loss detected (of 26)** | **External: over-called (of 74)** |
| --- | --- | --- | --- | --- | --- | --- | --- | --- |
| 0.001 | 0.772 | 60.2 | 82.9 | 0.970 / 0.987 | 0.739 | 2.62 | 23 | 15 |
| 0.005 | 0.553 | 69.1 | 86.2 | 0.979 / 0.985 | 0.782 | 2.52 | 26 | 15 |
| 0.01 | 0.488 | 72.4 | 88.6 | 0.982 / 0.985 | 0.801 | 2.49 | 26 | 15 |
| 0.02 | 0.463 | 72.4 | 90.2 | 0.983 / 0.985 | 0.818 | 2.44 | 26 | 15 |
| 0.03 | 0.480 | 70.7 | 90.2 | 0.984 / 0.984 | 0.828 | 2.42 | 26 | 15 |
| 0.05 (selected) | 0.439 | 74.0 | 91.1 | 0.985 / 0.984 | 0.835 | 2.38 | 26 | 15 |
| 0.1 | 0.463 | 72.4 | 90.2 | 0.986 / 0.981 | 0.842 | 2.39 | 26 | 17 |
| 0.15 | 0.488 | 70.7 | 88.6 | 0.987 / 0.980 | 0.837 | 2.42 | 26 | 18 |
| 0.2 | 0.553 | 68.3 | 87.0 | 0.987 / 0.976 | 0.842 | 2.42 | 26 | 20 |
| 0.25 | 0.699 | 58.5 | 84.6 | 0.987 / 0.970 | 0.846 | 2.44 | 26 | 25 |
| 0.3 | 0.805 | 52.8 | 80.5 | 0.987 / 0.966 | 0.854 | 2.43 | 26 | 32 |
| 0.35 | 0.992 | 43.1 | 76.4 | 0.987 / 0.958 | 0.847 | 2.61 | 26 | 43 |
| 0.4 | 1.130 | 37.4 | 71.5 | 0.987 / 0.951 | 0.840 | 2.71 | 26 | 48 |
| 0.45 | 1.325 | 30.9 | 65.9 | 0.987 / 0.943 | 0.823 | 2.96 | 26 | 53 |
| 0.5 | 1.626 | 23.6 | 55.3 | 0.988 / 0.931 | 0.805 | 3.14 | 26 | 57 |
| 0.55 | 1.992 | 13.8 | 47.2 | 0.988 / 0.917 | 0.780 | 3.44 | 26 | 66 |
| 0.6 | 2.561 | 8.9 | 33.3 | 0.989 / 0.894 | 0.754 | 3.77 | 26 | 69 |
| 0.65 | 3.220 | 4.1 | 21.1 | 0.990 / 0.871 | 0.718 | 4.19 | 26 | 71 |
| 0.7 | 4.293 | 1.6 | 11.4 | 0.990 / 0.828 | 0.640 | 5.11 | 26 | 72 |
| 0.75 | 6.350 | 0.0 | 0.8 | 0.991 / 0.751 | 0.509 | 6.87 | 26 | 74 |
| 0.8 | 10.382 | 0.0 | 0.0 | 0.993 / 0.598 | 0.287 | 10.19 | 26 | 74 |
| 0.85 | 16.081 | 0.0 | 0.0 | 0.993 / 0.381 | 0.107 | 14.68 | 26 | 74 |
| 0.9 | 23.293 | 0.0 | 0.0 | 0.989 / 0.107 | 0.018 | 20.06 | 26 | 74 |

**Table S8. Derivation of the three mutually exclusive tooth-status classes for the 705 disease-annotated DENTEX 2023 radiographs (20,699 tooth boxes). Where a tooth carried both a restoration and a caries annotation, restoration status took precedence. The restoration list was compiled by one study dentist (restored versus not restored only; no restoration types; no protocol for implants or prosthetic units) and its reliability was not formally assessed.**

| **Source annotation** | **Boxes** | **Assigned class** |
| --- | --- | --- |
| DENTEX caries | 2,189 | Caries (1,682); Filled (507, restored teeth) |
| DENTEX deep caries | 578 | Caries (481); Filled (97, restored teeth) |
| DENTEX periapical lesion | 158 | Filled (87, restored teeth); Healthy (71) |
| DENTEX impacted tooth | 604 | Healthy (604) |
| Sound teeth (per-tooth enumeration layer), restored per the external list | 2,272 | Filled (2,272) |
| Sound teeth (per-tooth enumeration layer), remaining | 14,898 | Healthy (14,898) |
| Total | 20,699 | Healthy 15,573; Caries 2,163; Filled 2,963 |
